# Who Is to Standardise Drugs?

**Published:** 1900-08-25

**Authors:** 


### WHO IS TO STANDARDISE DRUGS?

As we have pointed out in former articles, there is a growing desire that various drugs, the analysis of which by chemical means is difficult, and in some cases impracticable, should be standardised in terms of their physiological activity as shown by their action on the lower animals. There are, moreover, certain "drugs" if we may so call them, the anti-serums or so-called antitoxins, the strength of which can be ascertained in no other way than by such experiments on animals, a matter of great importance in view of the fact that unless their strength, and consequently their dose, be known they lose much of their utility for purposes of treatment. Under these circumstances one need not be surprised to find that a firm of manufacturing chemists should have applied to the Home Secretary for a licence under the Vivisection Act for the performance of the necessary experiments at their laboratories. The Home Office, on receiving this application, consulted the Royal Colleges of Physicians and of Surgeons on the subject, and the committee to which the matter was referred have reported to the effect (1) that with certain remedies, containing active agents which cannot be estimated by any known chemical processes, experiments on animals (usually rabbits, guinea-pigs, or mice) are absolutely necessary in order that their therapeutic strength may be ascertained. This especially applies to the anti-serums, since, for example, in the preparation of anti-diphtheritic serum different horses treated in the same way yield serum of different strengths, and it is pointed out that an unstandardised antitoxin is a source of danger. (2) But when it came to the question whether it would be desirable to commit to the manufacturing chemists the work of carrying out the experiments necessary for the standardising of their preparations, the committee thought that this would be highly undesirable. Among other reasons they pointed out that if such licences were issued it would be difficult to determine which firms should be permitted to make such experiments, especially in view of the fact that the possession of such a licence would be of value from a commercial point of view; and that experiments made for the purposes of trade would not have such an independent character as the purely scientific and humane experiments in pathology, physiology, and therapeutics, which are carried on at present under the Act. (3) The committee considered, however, that such standardisation of these remedies was absolutely essential for the public welfare, and thought that the antitoxic serums might be tested and certified by some responsible authority, either by the Government directly, in a State laboratory, or by some public body or bodies with the sanction of the Government. They further thought that such authoritative standardisation would carry more weight with the profession and the public than if it were left to the several manufacturers.

